# Peritoneal regression grading score (PRGS) in peritoneal metastasis: how many biopsies should be examined?

**DOI:** 10.1515/pp-2022-0118

**Published:** 2022-09-26

**Authors:** Wiebke Solass, Christoph Meisner, Florian Kurtz, Giorgi Nadiradze, Marc A. Reymond, Hans Bösmüller

**Affiliations:** Institute of Pathology, University Bern, Bern, Switzerland; National Center for Pleura and Peritoneum, Tuebingen, Germany; Institute of Pathology, Eberhard-Karls-University Tuebingen, Tuebingen, Germany; Institute for Clinical Epidemiology and Applied Biometry, Eberhard-Karls-University Tuebingen, Tuebingen, Germany; Deptartment of General and Transplant Surgery, Eberhard-Karls-University Tuebingen, Tuebingen, Germany

**Keywords:** antineoplastic agents, drugs administration routes, immunohistochemistry, intraperitoneal chemotherapy, peritoneal metastasis, peritoneal regression grading score (PRGS), pressurized intraperitoneal aerosol chemotherapy (PIPAC), tumor regression gastric cancer

## Abstract

**Objectives:**

The four-tied peritoneal regression grading score (PRGS) is increasingly used to evaluate the response of peritoneal metastases (PM) to chemotherapy. The minimal number of peritoneal biopsies needed for PRGS determination remains unclear.

**Methods:**

A prospective cohort of 89 PM patients treated with 210 pressurized intraperitoneal aerosol chemotherapy (PIPAC) cycles was investigated. Four biopsies from every abdominal quadrant were recommended. Histological tumor response was defined as a stable or decreasing mean PRGS between therapy cycles, progression increasing. We compared the diagnostic uncertainty induced by missing biopsies to the histological response.

**Results:**

A total of 49 patients had at least two PIPAC and were eligible for therapy response assessment. Mean PRGS decreased from 2.04 (CI 5–95% 1.85–2.27) to 1.79 (CI 5–95% 1.59–2.01), p=0.14, as a proof of therapy effectiveness. 35 (71.4%) patients had a stable or decreasing PRGS (therapy response), 14 (28.6%) a PRGS increase (disease progression). Histology showed agreement between four biopsies in 42/210 laparoscopies (20%), between ≥3 biopsies in 103 (49%), and between ≥2 biopsies in 169 laparoscopies (81%). Mean loss of information with one missing biopsy was 0.11 (95% CI=0.13) PRGS points, with two missing biopsies 0.18 (95% CI 0.21). In 9/49 patients (18.3%), the loss of information with one less biopsy exceeded the change in PRGS under therapy.

**Conclusions:**

A minimum of three biopsies is needed to diagnose PM progression with an accuracy superior to 80%. Missing biopsies often result in a false diagnosis of tumor progression.

## Introduction

Peritoneal metastasis (PM) is common in gastrointestinal and gynecological cancer patients, with an estimated incidence of one million cases/year worldwide [1]. Upon diagnosis, current therapy guidelines recommend palliative systemic chemotherapy or the best supportive care [2]. Due to the limited soft-tissue contrast and the mobility of the small bowel, CT depiction of small peritoneal tumors remains unsatisfactory [3, 4]. Against this framework, surgical oncologists increasingly use laparoscopy to assess the extent of tumor burden at initial diagnosis and to obtain tissue biopsies for therapy response assessment during treatment [5].

The peritoneal regression grading score (PRGS) was proposed in 2016 for reporting the regressive changes under palliative chemotherapy in PM of gastrointestinal and gynecological origin [6]. The PRGS considers two main tumor regression criteria: partial or complete disappearance of malignant cells and fibrous or fibro-inflammatory tissue replacement. It also includes acellular mucin pools and infarct-like necrosis (ILN) as additional features of response [6]. PRGS was shown to be reproducible for assessing the response to intraperitoneal chemotherapy in PM [6]. The best reproducibility was achieved by the mean, not the maximal PRGS.

In the meantime, multiple clinical trials and cohort studies used the PRGS as a surrogate outcome criterion in intraperitoneal chemotherapy. These trials included ovarian cancer [7], gastric cancer [8], colorectal cancer [9], hepatobiliary-pancreatic cancer, and various histologies [10–14]. Combining the highest PRGS with peritoneal cytology (so-called CPI) was independently associated with worse overall and progression-free survival [15]. This finding follows the initial proposal of the PRGS authors, who recommended sampling peritoneal fluid for cytological analysis in the presence of tumor scarring or the absence of macroscopic peritoneal lesions [6].

However, different peritoneal biopsies in other locations might deliver different PRGS scores due to tumor clonality, cell cycle phases, or various expositions to chemotherapy. Moreover, due to intraabdominal adhesions, it is not always possible to take biopsies in every four abdominal quadrants, and the minimal number of biopsies needed to determine PRGS remains unknown.

We aimed to examine the minimum number of biopsies required to obtain reliable information on therapy response using the PRGS score and provide a corresponding, evidence-based recommendation.

## Materials and methods

### Study design

This study is a retrospective analysis of samples and information collected in a prospective registry of consecutive patients treated with intraperitoneal chemotherapy (as Pressurized IntraPeritoneal Aerosol, Chemotherapy, PIPAC) combined or not with systemic chemotherapy at our institution between 1.7.2016 and 31.12.2018.

### Ethical and regulatory framework

The international PIPAC patient registry (NCT03210298) was approved by the Ethics Committee, Ruhr-University Bochum (Reference 15–5,280). The registry was hosted by an independent quality control organization (AnInstitut für Qualitätssicherung in der Operativen Medizin gGmbH, Otto-von-Guericke Universität Magdeburg). Each patient gave written informed consent both for the therapeutic procedures and for data storage management and analysis.

### Peritoneal biopsy specimens

All patients included in the present study underwent at least two PIPAC procedures. We used 5-mm laparoscopic forceps (ClickLine Biops^®^, Karl Storz, Tuttlingen, Germany) for taking biopsies from tumor suspect areas in every four abdominal quadrants. However, only one, two, or three peritoneal biopsies were taken in some instances because of adhesions preventing access to some abdominal quadrants.

### Preanalytic steps

Peritoneal biopsies were fixed in 10% buffered formalin for 24–48 h. Then, samples were embedded in paraffin using a controlled temperature. Two series of three 2 µm thick step sections from each biopsy were stained with hematoxylin and eosin (H&E) at the Institute of Pathology and Neuropathology, University Hospital Tübingen, Germany.

### Determination of PRGS

The histological tumor response was determined by an independent pathologist (specialized in the field of gastrointestinal and gynecological malignancies) according to the four-tied peritoneal regression grading score (PRGS), as described elsewhere [6]. PRGS scoring was retrieved from clinical records. A reference pathologist did not verify the scoring since this would not reflect clinical practice.

### Definition of tumor response vs. progression

Clinical decisions in palliative oncology are based on objective tumor regression vs. progression. We defined the PRGS histological response criteria in analogy with RECIST 1.1 radiological tumor response criteria (Table 1). Histological assessment of tumor response requires comparing at least two consecutive biopsies so that only patients with ≥2 therapy cycles are eligible for such evaluation.

**Table 1:** Definition of histological tumor response/progression with the peritoneal regression grading score (PRGS).

		RECIST 1.1	PRGS
**Progression**	Progression of disease (PD)	≥20% increase in the sum of diameters	Increase of mean PRGS
**Tumor response**	Stable disease (SD)	Any case that not qualify for CR, PR, or PD	Mean PRGS stable
	Partial response (PR)	≥30% decrease in the sum of diameters	Decrease of mean PRGS
	Complete response (CR)	The disappearance of all measurable lesions	No vital tumor cells (PRGS 1)

### Study flow

**Figure 1:**
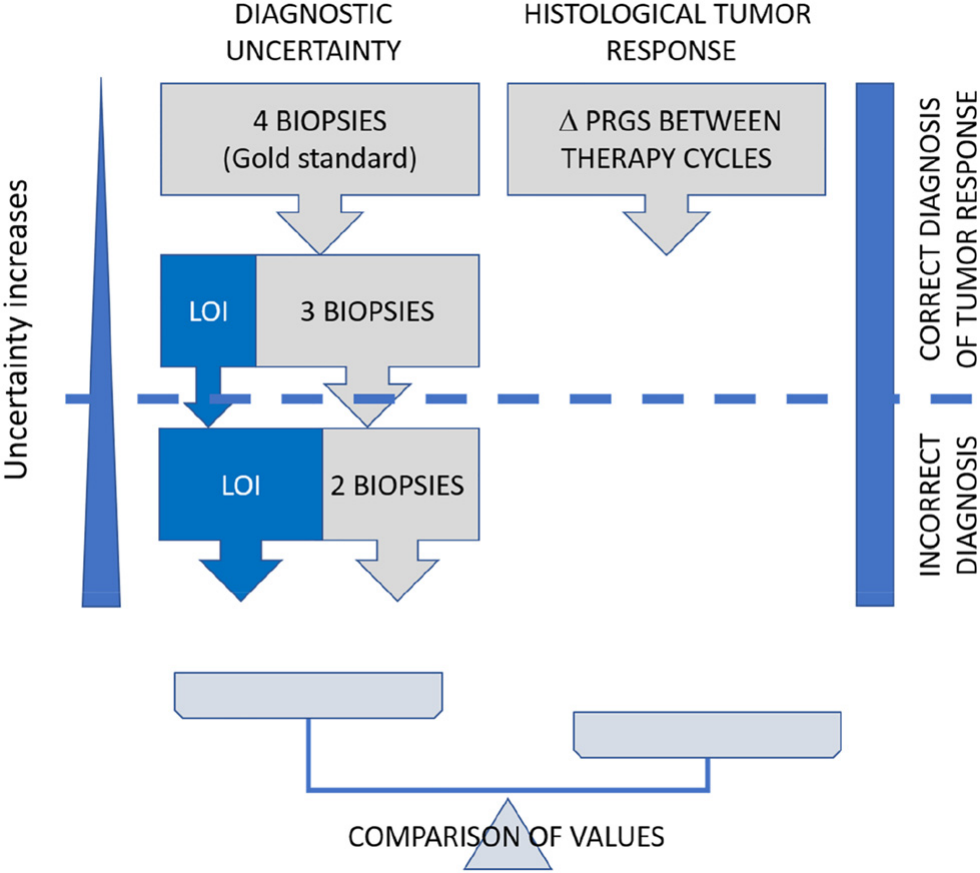
Study flow. We determined the mean PRGS from all four peritoneal biopsies, and the loss of information (LOI) associated with one and two biopsies missing (left part). In parallel, the mean difference in PRGS between consecutive chemotherapy cycles was documented (right part). When the loss of information outweighed the 95% CI of the observed difference, the diagnosis of regression vs. progression was considered false. Δ PRGS = difference in mean PRGS between consecutive therapy cycles.

**Figure 2:**
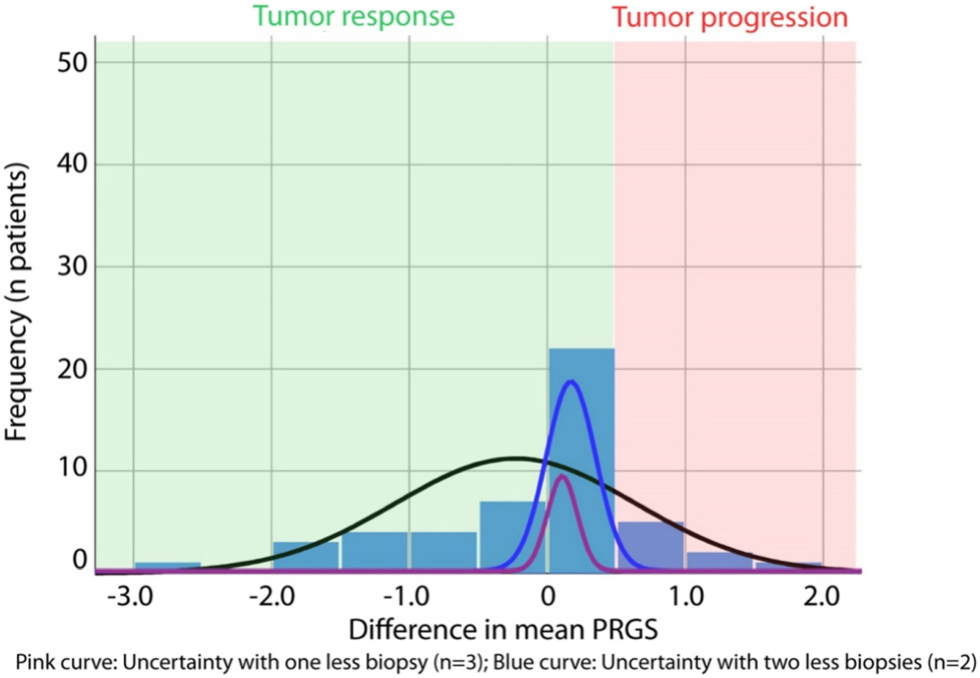
Histological tumor response and informational loss induced by missing biopsies. The tumor response is measured as the absolute difference of the mean PRGS between two therapy cycles. Progression of disease (PD) is defined by an increase in the mean PRGS (red area). Tumor response (green area) is defined by an unchanged PRGS (stable disease, SD), a decrease in the mean PRGS (partial response, PR), or the absence of vital tumor cells in all biopsies (PRGS 1, complete response, CR). The pink curve shows the diagnostic uncertainty induced by one missing biopsies (= only three biopsies available), the blue curve by two missing biopsies (= only two biopsies available). Diagnosis of tumor progression is wrong when the diagnostic uncertainty is superior to the difference in the mean PRGS.

The study flow is displayed in Figure 1. To quantify the loss of information caused by missing biopsies, it was first necessary to decide on a reference value (gold standard). In the absence of an external comparator, we chose the gold standard to be the mean PRGS in all patients, where results from four biopsies from all abdominal quadrants were available. The loss of information was calculated by excluding one, two, and three biopsies. All combinations were tested, and the mean was calculated. In parallel, the mean difference in PRGS between consecutive chemotherapy cycles was documented. When the loss of information outweighed 95% CI of the absolute difference in PRGS observed between repeated biopsies, the diagnosis of regression vs. progression was considered false (Figure 2).

### Statistical analysis

#### Primary endpoint

The primary endpoint was the agreement in PRGS between different biopsies, expressed as a percentage of identical PRGS.

### Secondary endpoint

The secondary endpoint was the loss of information, defined as the difference in mean PRGS points in the scenario where only three, respectively, two biopsies are examined, as compared to four biopsies.

### Clinical decision

In clinical practice, the decision between tumor response vs. progression is determined based on the difference in the mean PRGS between consecutive PIPAC cycles. We thus examined the number of individual cases where the loss of information induced by missing biopsies would have changed this decision. We defined the cut-off as the percentile 95% of the deviation in the absolute mean PRGS measured for only three, respectively, two biopsies in all patients available. Using this cut-off, we accepted to miss 5% of patients with an incorrect clinical decision.

### Statistical hypothesis

The H0 hypothesis for the primary endpoint was a complete agreement in the PRGS between individual biopsies. The H0 hypothesis for the secondary endpoint was an identical mean PRGS/laparoscopy, independently of the number of biopsies available.

Values are given as means or medians, where appropriate. Comparisons between groups were performed using nonparametric tests. We used SPSS 24 for Windows (SPSS Inc., Chicago, IL, USA) for statistical analysis.

## Results

A cohort of 89 patients with PM of various origins treated with PIPAC was available for analysis (Table 2). Altogether, 641 biopsies from 210 laparoscopies were available for histological examination. Fifty-four patients had at least two laparoscopies (= PIPAC cycles) and were eligible for tumor response assessment.

**Table 2:** Tumor histology and therapy cycles.

		Number	%
Tumor type	Gastric	31	34.8
	Hepatobiliary-pancreatic	17	19.1
	Colorectal	12	13.5
	Ovarian	9	10.1
	Appendiceal	6	6.7
	Pseudomyxoma peritonei	4	4.5
	Peritoneal mesothelioma	2	2.2
	Other origins	8	9.0

Number of patients		89	100%

Therapy cycles	1	89	42.4
	2	54	25.7
	3	31	14.8
	4	19	9.0
	5	11	5.2
	≥6	76	2.9

Number of laparoscopies		210	100%

### Agreement between peritoneal biopsies

Table 3 summarizes the information on the agreement between peritoneal biopsies. Out of 210 laparoscopies, 168 (81.4%) showed agreement in ≥ two biopsies, 103 (49.0%) in ≥ three biopsies, and 39 (18.6%) in all four biopsies. Thus, there was significant heterogeneity between PRGS scores obtained from biopsies in different localizations.

**Table 3:** Number of biopsies available and PRGS agreement between biopsies.

	Frequency	Percentage	Cumulative %
Total of biopsies taken			

1	30	14.3	14.3
2	29	13.8	28.1
3	51	24.3	52.4
4	100	47.6	100
n Laparoscopies	210	100	100

PRGS agreement between biopsies			

1	42	20.0	20.0
2	65	31.0	51.0
3	64	30.5	81.4
4	39	18.6	100
n Laparoscopies	210	100	100

### Loss of information depending on the number of biopsies

We then examined the loss of PRGS information induced by missing biopsies. For this purpose, we focused the analysis on a smaller cohort of patients with all four biopsies available (n=49 patients, 196 biopsies in total). In these 49 patients, we determined the difference explained by one, respectively, two fewer biopsies by the following method: we calculated the mean PRGS for each possible combination of three biopsies and compared the results with the gold standard (four biopsies). We repeated the same process with only two biopsies. The mean absolute difference from the gold standard was 0.105 (95% CI: 0.085–0.126) PRGS points for three biopsies and increased to 0.173 (95% CI: 0.139–0.207) with two biopsies. This difference directly quantifies the loss of information/additional uncertainty with one, respectively, two lacking biopsies vs. all four biopsies.

### Objective histological assessment of tumor response

A subgroup of 54 patients had at least two PIPAC cycles and was eligible for tumor response assessment. Five of these patients were excluded because only a local peritonectomy was available. The remaining 49 patients with at least two PIPAC cycles were considered eligible for histological tumor response assessment. We defined the PRGS histological response criteria in analogy with RECIST 1.1 radiological tumor response criteria (see Table 1) [16]. According to the above criteria, 35 (71%) patients had a tumor response, 14 (29%) a tumor progression. As determined by the mean difference between the first and second therapy cycle, the absolute tumor response was 0.588 (CI 5–95%: 0.504–0.854) PRGS points.

### Influence of missing biopsies on the clinical decision

From the above data, it was possible to compare the loss of information (diagnostic uncertainty) with the difference of mean PRGS between therapy cycles (diagnosis of response, respectively progression). We determined the number of patients where the loss of information due to one, respectively, two lacking biopsies outweighed the observed difference between successive therapy cycles (Figure 1). One fewer biopsy would have led to a false diagnosis of tumor progression in 9/49 patients with two fewer biopsies, two additional patients would have been misdiagnosed. Thus, it is possible to calculate the positive predictive value (PPV) and negative predictive value (NPV) as well as the accuracy of PRGS for tumor progression with four, three, and two biopsies in our set of patients. These values are summarized in Table 4.

**Table 4:** Performance metrics of PRGS score for prediction of tumor progression with one or two biopsies missing.

Three biopsies available		Gold standard (4 biopsies)		
		Response	Progression	Total
Test	Response	26 (TN)	0 (FN)	26
	Progression	9 (FP)	14 (TP)	23
	Total	35	14	49
Sensitivity for tumor progression				100% (95% CI 76,8–100%)
Specificity for tumor progression				74.3% (95% CI 56.4–87.5%)
Positive predictive value for tumor progression, PPV				100%
Negative predictive value for tumor progression, NPV				60.9% (95% CI 47.0–73.2%)
Accuracy for tumor progression				81.6% (95% CI 68.0–91.2%)

Two biopsies available		Gold standard (4 biopsies)		
		Response	Progression	Total
Test	Response	25 (TN)	1 (FN)	26
	Progression	10 (FP)	13 (TP)	23
	Total	35	14	49
Sensitivity for tumor progression				92.8% (95% CI 53.7–85.4%)
Specificity for tumor progression				71.4% (95% CI 66.1–99.8%)
Positive predictive value for tumor progression, PPV				96.2% (95% CI 78.9–99.4%)
Negative predictive value for tumor progression, NPV				56.2% (95% CI 43.0%–69.1%)
Accuracy for tumor progression				77.6% (95% CI 63.4–88.2%)

TP, true positive; FP, false positive; TN, true negative; FN, false negative.

### Role of an additional local peritonectomy

In the original PRGS proposal [6], the authors recommended performing a centimetric local peritonectomy to increase the chances of detecting minimal tumor residues. Out of 18 patients with tumor-free biopsies from all four abdominal quadrants, an additional centimetric peritonectomy was performed in 15 patients. Histological analysis of the peritonectomy sample did not show tumor cells in any of these patients. Thus, no useful additional information was provided by the local peritonectomy in this cohort, and peritonectomy was futile in this cohort.

## Discussion

The lack of a radiological gold standard highlights the challenges for assessing tumor response in PM patients. A histological score, the PRGS, was developed to address this challenge. PRGS is a simple four-tied system [6], is reproducible [17], and has been used in independent cohorts on an international level [7]. PRGS is tailored to peritoneal metastasis, taking into account their frequent mucinous nature [6]. In analogy to the revised RECIST guideline [18], histological PM progression was defined as an increase in mean PRGS between therapy cycles and response to therapy as a decrease or stable mean PRGS [13].

This study aimed to examine the minimum number of peritoneal biopsies required to obtain a reliable diagnosis of tumor progression with PRGS and provide a corresponding, evidence-based recommendation. For this purpose, we determined the information loss per missing biopsy and compared this information loss with the difference in PRGS observed between therapy cycles (histological tumor response). We calculated that a minimum of three peritoneal biopsies is required to reach at least 80% accuracy for determining tumor progression using PRGS. Against this framework, it is essential to note that accuracy is not recommended as a quality metric for diagnostic tests. The cut-off of 80% used for clinical significance is arbitrary. Specificity and sensitivity metrics show that missing biopsies often result in false-positive results and, thus, false diagnosis of non-response. This knowledge is essential for proper clinical decision-making, and pathologists should be cautious about diagnosing tumor progression with less than four peritoneal biopsies.

We decided to use the mean PRGS obtained from all biopsies and not the maximal PRGS from a single biopsy to prevent loss of information. Dropping three out of four biopsies (=75% of the information available) might cause an unacceptable risk of bias. Our preference is in line with the prior art in regression grading, recommending considering all sections of the tumor bed rather than the worst section (highest grade) [19].

Prior work has highlighted that spatial and temporal tumor heterogeneity increases during disease progression, probably because of clonal selection [20]. Tumor heterogeneity is a significant reason for chemoresistance [21], which in turn worsens patients’ prognosis. Therefore, since PM is a manifestation of advanced disease, consideration of tumor heterogeneity appears essential to evaluating tumor response to chemotherapy and prognostic assessment. Against this framework, there is an obvious need for a uniform sampling method for determining tumor regression grading [19]. In particular, a minimal number of biopsies needs to be determined that consider tumor heterogeneity between sampling locations. To our knowledge, this is the first study showing that the histological analysis of a single or even two tumor biopsies does not provide accurate information on the response of PM to chemotherapy.

The current lack of evidence on the number of biopsies needed to classify histological tumor regression is in sharp contrast with the binding recommendations of the UICC on the minimal number of lymph nodes needed to determine the pN category in tumor staging. When a minimal number of lymph nodes are not available for microscopic analysis and determination of vital tumor cells, then the pN category cannot be safely determined, and the case might be classified pNx. In analogy, based on our data, we recommend ranking PRGS as undetermined (“PRGSx”) in any case where a minimum of three peritoneal biopsies is not available.

In contrast to other studies [22], no external comparator such as, for example, a surgical specimen was available for this study. RECIST criteria were not considered as a reliable external comparator either, since they are, to our knowledge, never been validated for assessing the response of peritoneal metastasis to chemotherapy. As an alternative, we decided to use an internal comparator as the gold standard and calculated for this purpose the mean PRGS in patients with histological information from all abdominal quadrants. Then, it was easy to calculate the loss of information per missing biopsy and correlate this value with the difference of PRGS observed between therapy cycles.

In summary, this study indicates that the use of PRGS may address the need for reliable, objective, reproducible tumor response assessment in patients with PM. PRGS adds valuable information to the RECIST 1.1 criteria, which can barely reflect intraperitoneal tumor heterogeneity. Notably, this is the first study to our knowledge to investigate the minimal number of biopsies required to assess histological tumor response under palliative chemotherapy. Our results provide compelling evidence for intraperitoneal tumor heterogeneity and various degrees of tumor response depending on tumor location. Thus, PRGS appears effective for assessing the response of PM to chemotherapy.

Future research should follow three avenues. First, prospective cohort studies are needed to validate PRGS as a predictive or prognostic parameter in PM of various origins. Second, the potential role of additional staining in PRGS1 (no vital tumor cells) cases should be investigated. Last but not least, molecular parameters might be integrated into the PRGS, paving the way for individualized therapy of patients with PM and improving the prognosis of this challenging patient population.
